# A revised Fisher model on analysis of quantitative trait loci with multiple alleles

**DOI:** 10.3389/fgene.2014.00328

**Published:** 2014-09-25

**Authors:** Tao Wang

**Affiliations:** Division of Biostatistics, Institute for Health and Society, Medical College of WisconsinMilwaukee, WI, USA

**Keywords:** Fisher's genetic model, genetic variance components, general two-allele model, general multi-allele model, orthogonality, least square approach, average allelic effects and interactions

## Abstract

Zeng et al. (2005) proposed a general two-allele (G2A) model to model bi-allelic quantitative trait loci (QTL). Comparing with the classical Fisher model, the G2A model can avoid using redundant parameters and be fitted directly using standard least square (LS) approach. In this study, we further extend the G2A model to general multi-allele (GMA) model. First, we propose a one-locus GMA model for phase known genotypes based on modeling the inheritance of paternal and maternal alleles. Next, we develop a one-locus GMA model for phase unknown genotypes by treating it as a special case of the phase known one-locus GMA model. Thirdly, we extend the one-locus GMA models to multiple loci. We discuss how the genetic variance components can be analyzed using these GMA models in equilibrium as well as disequilibrium populations. Finally, we apply the GMA model to a published experimental data set.

## 1. Introduction

Currently there are two types of statistical genetic models that are commonly used in genetic analysis of quantitative traits. One is the F_∞_ type models that concentrate on direct modeling of the expected genotypic values at targeted quantitative trait loci (QTL) or genetic markers and association testing for various allelic effects and interactions (Fisher, 1918; Cheverud, 2000; Hansen and Wagner, 2001; Wang, 2011). Another is Fisher's analysis of variance (ANOVA) models that focus on assessing variations contributed by some genetic components (i.e., grouped allelic effects or allelic interactions) at targeted QTL or genetic markers (Fisher, 1918; Cockerham, 1954, 1963; Kempthorne, 1969; Weir and Cockerham, 1977; Wang and Zeng, 2006). A considerable amount of discussion has been made about the distinction between these two different types of genetic models (Zeng et al., 2005; Álvarez-Castro and Carlborg, 2007; Yang and Álvarez-Castro, 2008; Wang and Zeng, 2009). More recently, a comprehensive review of various genetic models was also given in Álvarez-Castro (2012).

In genetic association studies, we are often interested in direct comparison of the expected genotypic values at certain QTL or marker loci. The *F*_∞_ models are appealing in this setting due to their simplicity in interpretation of their model parameters, which are often referred as the fixed genetic effects such as the additive and dominance effects or the allelic effects and allelic interactions in terms of the expected genotypic values. By applying the *F*_∞_ models, we can compare the expected genotypic values via hypothesis tests on various fixed genetic effects. However, as pointed out in Wang and Zeng (2009), the *p*-value based association tests on these fixed genetic effects could highly depend upon the regression model, the distribution assumption and the available sample size. Besides, a statistically significant genetic effect with a small enough *p*-value may not necessarily imply a clinically important finding. On the other hand, the Fisher type models allow us to assess the genetic variations contributed by certain genetic components to the overall variation of a quantitative trait. By definition, these genetic variance components do not depend on the sample size, and they can provide additional information on better understanding the genetic etiology and assessing for clinical importance. Nonetheless, both the F_∞_ and the Fisher type models form basis in the analysis of quantitative traits. They provide different perspectives in assessing the genetic effects of QTL or genetic markers.

The basic genetic model on assessing the genetic variance components was first proposed by Fisher (1918). Cockerham (1954, 1963) extended Fisher's one-locus model to two bi-allelic QTL with a particular focus on epistatic variance components. Kempthorne (1954, 1957) further extended the two-locus model to multiple alleles. Wang and Zeng (2006) also explored the Fisher type multi-allele two-locus model on partition of the genotypic variance in the presence of linkage disequilibrium (LD). With their model parameters referred as the average effect of the gene substitution (see Falconer and Mackay, 1996) or the average allelic effects and interactions (Wang and Zeng, 2009), these classical Fisher type models often contain constraints on their model parameters due to an over-parameterization of the expected genotypic values. These constraints could make it difficult to fit this type of models using standard least square (LS) regression approach. To avoid this problem, under a regression model framework, Zeng et al. (2005) proposed a general two-allele (G2A) model for variance component analysis on bi-allelic QTL. The G2A model retains the nice property of the classical Fisher's model on orthogonal partition of the genotypic variance in equilibrium populations. Meanwhile, without using redundant parameters, the G2A model can be fitted directly using the standard LS approach. Wang and Zeng (2009) further explored the origin of the G2A model and clarified its theoretical basis on pertaining the orthogonal partition of the genotypic variance in equilibrium populations. Álvarez-Castro and Carlborg (2007) also proposed a different way for re-parameterization of the expected genotypic values, which the authors referred as the natural and orthogonal interactions (NOIA) model. It appears that the NOIA model first defines the genetic effects in terms of the expected genotypic values, and then derives the design matrix for the genetic effects via an inverse of the matrix. Though the matrix formulation is helpful for facilitating the transformation between different parameterizations, making inverse of the matrix to construct the design matrix is difficult to implement analytically. For multiple QTL, how to define various genetic effects in terms of the expected genotypic values could also be a challenge especially when locus-by-locus interactions are involved. Besides, the NOIA model assumes that the design matrix is of full rank, which makes it unsuitable for reduced re-parameterization of the genotypic values. Currently, no NOIA model has been proposed for reduced re-parameterization of the expected genotypic values, or for multiple QTL in the presence of LD.

In this study, we further extend the G2A model to QTL with multiple alleles and multiple loci. In bi-allelic case, only one additive effect and one dominance effect are needed at each locus, and the locus-by-locus interactions can be easily included for constructing a full re-parameterization of the genotypic values. For one QTL with multiple alleles, how to define the dominance effects for various allelic interactions is not straightforward especially when phases of its genotypes are unknown. The extension to multiple loci is also cumbersome by the much more complex structure of locus-by-locus interactions. How to present the model and define various genetic variance components are not trivial tasks. To construct one-locus general multi-allele (GMA) model, we overcome the phase problem by appropriately merging the paternal and maternal allelic effects and allelic interactions in the phase-known situation. Typically, with phase unknown genotypes at a locus, we may have to assume that the paternal and maternal alleles have the same frequencies and contribute the same genetic effects so that we could merge them without distinguishing their parental origins. With phase-known genotypes, we can further break down the additive variance component into paternal and maternal variance components. For multiple QTL with multiple alleles, we develop concise expressions for constructing multi-locus GMA models and defining various genetic components. Explicit formulas for calculating various genetic variance components in equilibrium population are also derived.

The structure of this manuscript is organized as the following. First, we consider one multi-allele QTL with phase known genotypes. Following the same strategy as adopted in Wang and Zeng (2009), we start by introducing indicator variables that describe the inheritance of paternal and maternal alleles. Then we make mean corrections on these indicator variables and build one-locus GMA model based on the mean-corrected index variables. Next, we consider one QTL with phase unknown genotypes. We construct a one-locus GMA model for unphased genotypes by appropriately combining the paternal and maternal allelic effects in the phase known one-locus GMA model. We derive formulas on partitioning the genotypic variance into the additive and dominance variance components under Hardy-Weinberg equilibrium (HWE) as well as in Hardy-Weinberg disequilibrium (HWD) for both the phase-known and phase-unknown one-locus GMA models. Thirdly, we extend the one-locus GMA models to multiple loci with either phase known or phase unknown genotypes. Based on these multi-locus GMA models, we describe how the various genetic components can be defined. An orthogonal partition on the genotypic variance in an equilibrium population is also presented in both the phase known and unknown cases. In addition, we discuss how to construct the reduced multi-locus GMA models in practice. The difference in using *F*_∞_ models and GMA models to build reduced models for the expected genotypic values is explored. Finally, we apply the GMA model to a published experimental data set.

## 2. Methods and results

The variation of a quantitative trait *Y* is usually assumed to be contributed by both genetic and environmental effects. Let *G* denote the true (unobservable) genotypic value from the joint contribution of all the genetic factors to the quantitative trait *Y*. Given genotypes at targeted QTL or genetic marker loci, we focus on assessing the variations (i.e., variances) contributed by the allelic effects and interactions of the QTL or marker loci to the total genotypic variance *V_G_*.

### 2.1. One-locus model for phase known

First, let us consider a single QTL with multiple alleles *A*_1_, …, *A_m_* (*m* ≥ 2). Suppose we know the parental origins of the alleles for observed genotypes. Then we can distinguish the paternal and maternal allelic effects separately. With *m* alleles, there are in total *m*^2^ possible phased genotypes: (*A_i_, A_j_*), *i, j* = 1, …, *m*, where *A_i_* and *A_j_* represent the paternal and maternal allele, respectively. Let *G^i^_j_* = *E*[*G*|*g* = (*A_i_, A_j_*)] denote the expected genotypic value for a phased genotype (*A_i_, A_j_*) in a study population. Then there are totally *m*^2^ possible expected genotypic values *G^i^_j_, i, j* = 1, …, *m*. To model these expected genotypic values, Fisher's classical one-locus model is given by

(1) $$G_{j}^{i}=\mu +\alpha^{i}+\alpha_{j}+\delta_{j}^{i},\;i,j=1,\ldots ,m,$$

where α^*i*^ (or α_*j*_) is the so-called *average* additive or main allelic effect of a paternal (or maternal) allele *A_i_* (or *A_j_*), and δ^*i*^_*j*_ is the *average* allelic interaction between a paternal allele *A_i_* and a maternal allele *A_j_*. The above model is a typical two-way ANOVA model with the paternal and maternal gametes being treated as two independent risk factors. Although the paternal and maternal gametes often share (but do not have to) the same set of alleles *A*_1_, …, *A_m_* at the QTL, it allows the paternal and maternal gametes to have different allele frequencies and allelic effects at the QTL.

Let *p*^*i*^ be the frequency of allele *A_i_* on paternal gametes $\left(\sum_{i\;=\;1}^{m}p^{i}=1\right)$, and *p_j_* be the frequency of allele *A_j_* on the maternal gametes $\left(\sum_{j\;=\;1}^{m}p_{j}=1\right)$. One nice feature of the Fisher model above is that it can assess the additive and dominance variance components *V_A_* and *V_D_*, which are defined as variations contributed by the additive allelic effects from the paternal and maternal alleles and the allelic interactions between the paternal or maternal alleles, respectively. For example, under HWE, it is well known that the total genotypic variance *V_G_* = ∑*_i,j_p^i^p_j_*(*G^i^_j_* − μ)^2^ has an orthogonal partition *V_G_* = *V_A_* + *V_D_*, where

$$V_{A}=\sum_{i}p^{i}{\left(\alpha^{i}\right)}^{2}+\sum_{j}p_{j}{\left(\alpha_{j}\right)}^{2}\;,\;\;\;V_{D}=\sum_{i,j}p^{i}p_{j}{\left(\delta_{j}^{i}\right)}^{2}.$$

Note that there are in total *m*^2^ + 2*m* + 1 = (*m* + 1)^2^ parameters being involved in Fisher model (1) including the intercept μ, which is more than the total number *m*^2^ of the expected genotypic values *G^i^_j_, i, j* = 1, …, *m*. As a result, not all the model parameters are estimable. To avoid this problem, some constraints on the model parameters are often required. It is usually assumed that all the genetic effects are averaged to zero over any index; i.e.,

(2) $$\sum_{i}p^{i}\alpha^{i}=0,\;\;\sum_{j}p_{j}\alpha_{j}=0,\;\;\sum_{i}p^{i}\delta_{j}^{i}=0,\;\;\sum_{j}p_{j}\delta_{j}^{i}=0.$$

With these constraints, Fisher (1918) showed that the least square estimates (LSE) of the model parameters are given by

$$\begin{array}{l} \mu =E(G),\;\alpha_{i}=G_{.}^{i}-G_{.}^{\;\cdot }\;,\;\alpha_{j}=G_{j}^{\;\cdot }-G_{.}^{\;\cdot }\;,\;\delta_{j}^{i}=G_{j}^{i}-G_{.}^{i}\\ \;-G_{j}^{\;\cdot }+G_{.}^{\;\cdot } \end{array}$$

where *G*^·^_._ = *E*(*G*), *G*^*i*^_._ = *E*[*G*|*g* = (*A_i_*, −)] and *G*^·^_*j*_ = *E*[*G*|*g* = (−, *A_j_*)]. In simple cases, we could estimate the model parameters using these formulas. In general, however, those “irregular” constraints in (2) make it difficult to fit model (1) using the standard LS approach via commonly used software such as SAS (SAS Institute INC, Raleigh, NC), especially when we need to adjust for certain environmental covariates.

Here we propose a way to get rid of the redundant parameters. Let us first introduce the following indicator variables that describe the transmission inheritance of the paternal and maternal alleles.

$$\begin{array}{l} \;z_{P_{i}}(g)=\{\begin{array}{l} 1,\text{ the paternal allele is }A_{i}\\ 0,\text{ the paternal allele is not }A_{i}\;, \end{array}\\ z_{M_{j}}(g)=\{\begin{array}{l} 1,\text{ the maternal allele is }A_{j}\\ 0,\text{ the maternal allele is not }A_{j}\;, \end{array} \end{array}$$

for *i, j* = 1, 2, …, *m* at the QTL. Next, using the same strategy as adopted in Wang and Zeng (2006), we further make mean corrections on these indicator variables *z_P_i__, z_M_j__* and define the following mean-corrected index variables

$$\begin{array}{l} \;x_{P_{i}}(g)=z_{P_{i}}(g)-E[z_{P_{i}}(g)]\\ \;=\{\begin{array}{l} 1-p^{i},\text{ the paternal allele is }A_{i}\\ -p^{i},\text{the paternal allele is not }A_{i}\;, \end{array}\\ x_{M_{j}}(g)=z_{M_{j}}(g)-E[z_{M_{j}}(g)]\\ \;=\{\begin{array}{l} 1-p_{j},\text{ the paternal allele is }A_{j}\\ -p_{j},\text{the paternal allele is not }A_{j}\;, \end{array} \end{array}$$

Then we can re-write the Fisher model (1) as

(3) $$\begin{array}{l} E(G|g)=\mu +\sum_{i\;=\;1}^{m}\alpha^{i}x_{P_{i}}(g)+\sum_{j\;=\;1}^{m}\alpha_{j}x_{M_{j}}(g)\\ \;+\sum_{i,j}\delta_{j}^{i}x_{P_{i}}(g)x_{M_{j}}(g), \end{array}$$

where *E*(*G*|*g*) denotes the expected genotypic value given a genotype *g* at the targeted QTL. As shown in Wang and Zeng (2006), the above model is simply a different representation of the Fisher model (1) with the same constraints being applied on the model parameters. However, as the HWD measurements can be quantified as covariances between those mean-corrected index variables *x_P_i__*'s and *x_M_j__*'s, this model expression can facilitate derivation on examining the genetic variance components under HWD.

Now, we further exclude the redundant parameters in model (3). For a diploid subject such as human being, his or her genotype at a locus on a pair of homologous chromosomes consists of two alleles with one from the father and the other one from the mother. Therefore, we always have $\sum_{i\;=\;1}^{m}z_{P_{i}}(g)\equiv 1$ and $\sum_{j\;=\;1}^{m}z_{M_{j}}(g)\equiv 1$. Thus, $x_{P_{m}}=-\sum_{i\;=\;1}^{m\;-\;1}x_{P_{i}}$ and $x_{M_{m}}=-\sum_{j\;=\;1}^{m\;-\;1}x_{M_{j}}$. So we can simply replace *x_P_m__* by $\left(-\sum_{i\;=\;1}^{m\;-\;1}x_{P_{i}}\right)$, and *x_M_m__* by $\left(-\sum_{j\;=\;1}^{m\;-\;1}x_{M_{j}}\right)$ in model (3), which leads to the following revised Fisher model

(4) $$\begin{array}{l} E(G|g)=\mu +\sum_{i\;=\;1}^{m\;-\;1}\alpha^{*i}x_{P_{i}}(g)+\sum_{j\;=\;1}^{m\;-\;1}\alpha_{*j}x_{M_{j}}(g)\\ \;+\sum_{i\;=\;1}^{m\;-\;1}\sum_{j\;=\;1}^{m\;-\;1}\delta_{*j}^{*i}x_{P_{i}}(g)x_{M_{j}}(g). \end{array}$$

Model (4) provides a full re-parameterization of the *m*^2^ expected genotypic values *G*^*i*^_*j*_, *i, j* = 1, …, *m*, without using redundant parameters. We refer it as a revised one-locus Fisher model or a general multi-allele (GMA) model. We also refer its model parameters α^*i^ (or α_*j_) as the average (additive) allelic effect of the paternal (or maternal) allele *A_i_* (or *A_j_*), and δ^*i^_*j_ the average allelic interaction between the paternal allele *A_i_* and maternal allele *A_j_*, with respect to the baseline allele *A_m_*. Here, we choose allele *A_m_* as the baseline allele but it could be any other allele as well. It is easy to show that, in terms of the parameters in the original Fisher model (1), we have α^*i^ = α^*i*^ − α^*m*^, α_*j_ = α_*j*_ − α_*m*_ and δ^*i^_*j_ = δ^*i*^_*j*_ − δ^*m*^_*j*_ − δ^*i*^_*m*_ + δ^*m*^_*m*_, for *i, j* = 1, …, *m*.

Model (4) retains most of the nice features of the original Fisher model (1) on partition of the genotypic variance. Based on this model, we have the genetic additive variance components $V_{A_{P}}=\text{Var}(\sum_{i\;=\;1}^{m\;-\;1}\alpha^{*i}x_{P_{i}})$ and $V_{A_{M}}=\text{Var}(\sum_{j\;=\;1}^{m\;-\;1}\alpha_{*j}x_{M_{j}})$, which denote variations contributed by the additive effects of the paternal and maternal alleles, respectively. The genetic dominant variance component $V_{D}=\text{Var}(\sum_{i\;=\;1}^{m\;-\;1}\sum_{i\;=\;1}^{m\;-\;1}\delta_{*j}^{*i}x_{P_{i}}x_{M_{j}})$, which accounts for a variation contributed by the interactions between paternal and maternal alleles in addition to the additive variance components. Under HWE, the paternal index variables {*x_P_i__, i* = 1, …, *m*} are independent of the maternal index variables {*x_M_j__, j* = 1, …, *m*}. Therefore, we have μ = *E*(*G*) and an orthogonal partition on the variance of the expected genotypic values: *V*(*E*(*G*|*g*)) = *V_A_P__* + *V_A_M__* + *V_D_*, where

$$\begin{array}{l} V_{A_{P}}=\sum_{i\;=\;1}^{m\;-\;1}p^{i}{\left(\alpha^{*i}\right)}^{2}-\left(\sum_{i\;=\;1}^{m\;-\;1}p^{i}\alpha^{*i}\right)2,\\ V_{A_{M}}=\sum_{j\;=\;1}^{m\;-\;1}p_{j}{\left(\alpha_{*j}\right)}^{2}-\left(\sum_{j\;=\;1}^{m\;-\;1}p_{j}\alpha_{*j}\right)2\;\;\\ \;V_{D}=\sum_{i,j\;=\;1}^{m\;-\;1}p^{i}p_{j}{\left(\delta_{*j}^{*i}\right)}^{2}-\sum_{i\;=\;1}^{m\;-\;1}p^{i}{\left(\sum_{j\;=\;1}^{m\;-\;1}p_{j}\delta_{*j}^{*i}\right)}^{2}\\ \;-\sum_{j\;=\;1}^{m\;-\;1}p_{j}{\left(\sum_{i\;=\;1}^{m\;-\;1}p^{i}\delta_{*j}^{*i}\right)}^{2}+{\left(\sum_{i,j\;=\;1}^{m\;-\;1}p^{i}p_{j}\delta_{*j}^{*i}\right)}^{2}. \end{array}$$

In HWD, the disequilibrium measurements can be captured by the covariances between the index variables *x_P_i__*'s and *x_M_j__*'s. In this case, we no longer have an orthogonal partition on the variance of the genotypic values for the additive and dominance variance components (see **Appendix A**). It should be pointed out that although the model parameters defined in model (4) depend upon the choice of the reference allele *A_m_*, the additive and dominant variance components *V_A_P__*, *V_A_M__*, *V_D_* as well as their covariance components do not depend on such a choice.

Note that the partition of the total genotypic variance *V_G_* based on the Fisher model (1) assumes that the genotypic values are completely determined by the QTL. In general, beyond the targeted QTL, the genotypic value *G* could also be affected by other unobserved QTL. In this case, the total genotypic variance *V_G_* = *V*(*E*(*G*|*g*)) + *E*(*V*(*G*|*g*)), where *V*(*G*|*g*) is a variation that cannot be explained by the targeted QTL. So the Fisher model (1), or its revised model (4), actually provides a partition on the variance *V*(*E*(*G*|*g*)) of the expected genotypic values at the targeted QTL instead of the total genotypic variance *V_G_*. In practice, given a random sample from the study population, if we ignore the genetic by environmental interaction, a quantitative trait can typically be expressed in a linear regression model form as

$$y_{k}=\beta z_{k}+E(G|g_{k})+\epsilon_{k},\;k=1,\ldots ,n,$$

where, for *k* = 1, …, *n*, *g_k_* is the observed QTL genotype of subject *k*, *z_k_* is a vector of subject *k* from some adjusted environmental covariates with fixed effects β, and ϵ_*k*_ is a model residual contributed by other environmental and genetic factors that cannot be captured by *z_k_* and *g_k_* at the targeted QTL. Assuming that ϵ_*k*_, *k* = 1, …, *n*, are independent and identically distributed (i.i.d) with a variance σ^2^_ϵ_, we have *V_y_* = *V*(*E*(*G*|*g*)) + σ^2^_ϵ_. To further partition *V*(*E*(*G*|*g*)) based on the QTL genotypes, we can first calculate the allele frequencies and compute the values of the index variables {*x_P_i__*(*g_k_*), *x_M_j__*(*g_k_*)} for each subject *k* = 1, …, *n*, in the sample. Then, by treating these index variables as fixed covariates, we can incorporate the GMA model (4) into the regression model above and fit the model using the standard LS approach. Based on the LSE ${\hat{\alpha }}^{*i}$, ${\hat{\alpha }}_{*j}$ and ${\hat{\delta }}_{*j}^{*i}$ of the model parameters, we can compute the additive and dominance genetic components $A_{P}(k)=\sum_{i\;=\;1}^{m\;-\;1}{\hat{\alpha }}^{*i}x_{P_{i}}(g_{k}),\;A_{M}(k)=\sum_{j\;=\;1}^{m\;-\;1}{\hat{\alpha }}_{*j}x_{M_{j}}(g_{k})$ and $D(k)=\sum_{i\;=\;1}^{m\;-\;1}\sum_{j\;=\;1}^{m\;-\;1}{\hat{\delta }}_{*j}^{*i}x_{P_{i}}(g_{k})x_{M_{j}}(g_{k})$, for *k* = 1, …, *n*. Finally, the genetic variance components *V_A_P__*, *V_A_M__* and *V_D_* can be estimated as the sample variances of {*A_P_*(*k*), *k* = 1, …, *n*}, {*A_M_*(*k*), *k* = 1, …, *n*}, and {*D*(*k*), *k* = 1, …, *n*}, respectively. Meanwhile, the genetic covariance components Cov(*A_P_, A_M_*), Cov(*A_P_, D*), and Cov(*A_M_, D*) can be estimated through the sample covariances

$$\begin{array}{l} \hat{\text{Cov}(A_{P},A_{M})}=\frac{1}{n}\sum_{k\;=\;1}^{n}(A_{P}(k)-{\bar{A}}_{P})(A_{M}(k)-{\bar{A}}_{M}),\\ \;\hat{\text{Cov}(A_{P},D)}=\frac{1}{n}\sum_{k\;=\;1}^{n}(A_{P}(k)-{\bar{A}}_{P})(D(k)-\bar{D}),\\ \;\hat{\text{Cov}(A_{M},D)}=\frac{1}{n}\sum_{k\;=\;1}^{n}(A_{M}(k)-{\bar{A}}_{M})(D(k)-\bar{D}), \end{array}$$

respectively, where ${\bar{A}}_{P}=\sum_{k\;=\;1}^{n}A_{P}(k)/n,\;{\bar{A}}_{M}=\sum_{k\;=\;1}^{n}A_{M}(k)/n$ and $\bar{D}=\sum_{k\;=\;1}^{n}D(k)/n$.

### 2.2. One-locus model for phase unknown

In this subsection, we consider modeling a multi-allele QTL with phase unknown genotypes—a more common situation in practice. As we cannot distinguish the parental origins of alleles in QTL genotypes, as usual, we assume that the paternal and maternal gametes share the same set of alleles with the same allele frequencies. Let *A*_1_, …, *A_m_* (*m* ≥ 2) denote the alleles at a target QTL or marker locus with allele frequencies *p_i_, i* = 1, …, *m*. Ignoring the phases, there are *m* possible homozygous genotypes *A_i_A_i_, i* = 1 …, *m*, and *m*(*m* − 1)/2 possible heterozygous genotypes *A_i_A_j_*, *i* ≠ *j*. We also assume that these alleles contribute the same genetic effects regardless of their parental origins, which implies that the expected genotypic values *G*_*ij*_ = *E*(*G*|*g* = *A_i_A_j_*) satisfy the symmetric property: *G_ij_* = *G_ji_*, for *i* ≠ *j*. So there are totally *m*(*m* + 1)/2 possible distinctive expected genotypic values *G_ij_*, *i, j* = 1, …, *m*. In this case, by treating the paternal and maternal gametes as two independent risk factors, the Fisher's ANOVA model for the expected genotypic values *G_ij_* can be written as

(5) $$G_{ij}=\mu +\alpha_{i}+\alpha_{j}+\delta_{ij},\;i,j=1,\ldots ,m,$$

where α_*i*_ is the average (additive) allelic effect of the paternal or maternal allele *A_i_* (*i* = 1, …, *m*), and δ_*ij*_ is the average allelic interaction between two alleles *A_i_* and *A_j_* (*i, j*, = 1, …, *m*). As pointed out in Wang (2011), the above model is different from the classical two-way ANOVA model in that the paternal and the maternal gametes share the same set of alleles and have the same allelic effects at the locus. To avoid the inestimability of model parameters in model (5) due to over-parameterization, the following constraints are often added on the model parameters

$$\sum_{i}p_{i}\alpha_{i}=0,\;\;\sum_{i}p_{i}\delta_{ij}=0.$$

From the symmetric property of *G_ij_*, we also assume δ_*jk*_'s are symmetric. Similarly, the above constraints together with the symmetry property of δ_*jk*_ make it difficult to fit model (5) using the standard LS approach.

Note that model (5) can be treated as a special case of model (1) or (3) with α^*i*^ = α_*i*_ and δ^*i*^_*j*_ = δ^*j*^_*i*_ for *i, j* = 1, …, *m*. To construct a similar GMA model for model (5), we can combine the term α^*i^*x_P_i__* with α_*i_*x_M_i__*, and δ^*i^_*j_*x_P_i__x_M_j__* with δ^*j^_*i_*x_P_j__x_M_i__* in model (4). By denoting α^*i^ = α_*i_ as α^*^_i_ for *i* = 1, …, *m* − 1, and δ^*j^_*i_ = δ^*i^_*j_ as δ^*^_ij_ for *i* ≤ *j*, we obtain the following GMA model

(6) $$\begin{array}{l} E(G|g)=\mu +\sum_{i\;=\;1}^{m\;-\;1}\alpha_{i}^{*}w_{i}(g)+\sum_{i\;=\;1}^{m\;-\;1}\delta_{ii}^{*}v_{ii}(g)\\ \;+\sum_{j\;=\;2}^{m\;-\;1}\sum_{i\;<\;j}\delta_{ij}^{*}v_{ij}(g), \end{array}$$

where, for *i* = 1, …, *m* − 1,

$$\begin{array}{l} w_{i}(g)=x_{P_{i}}+x_{M_{i}}=\{\begin{array}{l} 2(1-p_{i}),\text{if }g=A_{i}A_{i}\\ 1-2p_{i},\text{if }g=A_{i}A_{i}^{c}\\ -2p_{i},\text{if }g=A_{i}^{c}A_{i}^{c}\;, \end{array}\\ v_{ii}(g)=x_{P_{i}}x_{M_{i}}=\{\begin{array}{l} {\left(1-p_{i}\right)}^{2},\;i\text{f }g=A_{i}A_{i}\\ -p_{i}(1-p_{i}),\text{ if }g=A_{i}A_{i}^{c}\\ p_{i}^{2},\text{ if }g=A_{i}^{c}A_{i}^{c}\;, \end{array} \end{array}$$

and for *i* < *j*

$$v_{ij}(g)=x_{P_{i}}x_{M_{j}}+x_{P_{j}}x_{M_{i}}=\{\begin{array}{l} (1-p_{i})(1-p_{j})+p_{i}p_{j},\text{ if }g=A_{i}A_{j}\\ -2p_{j}(1-p_{i}),\text{ if }g=A_{i}A_{i}\\ -p_{j}(1-2p_{i}),\text{ if }g=A_{i}A_{ij}^{c}\\ -2p_{i}(1-p_{j}),\text{ if }g=A_{j}A_{j}\\ -p_{i}(1-2p_{j}),\text{ if }g=A_{j}A_{ij}^{c}\\ 2p_{i}p_{j},\text{ if }g=A_{ij}^{c}A_{ij}^{c}\;. \end{array}$$

Here *A*^*c*^_*ij*_ denotes an allele which is different from *A_i_* and *A_j_*. Note that the combined index variables *w_i_*(*g*), *v_ii_*(*g*), and *v_ij_*(*g*) above are well defined on unphased genotypes, although *x_P_i__, x_M_j__* are not. We refer them as genotype coding variables, and the model parameter α^*^_i_ as the average allelic effect of allele *A_i_* (*i* = 1, …, *m*), and δ^*^_ij_ as the average allelic interaction between two alleles *A_i_* and *A_j_* (*i, j*, = 1, …, *m*), with respect to the baseline allele *A_m_*. In terms of the parameters in the original model (5), we can show that α^*^_i_ = α_*i*_ − α_*m*_, for *i* = 1, …, *m* − 1; and δ^*^_ij_ = δ_*ij*_ − δ_*im*_ − δ_*jm*_ + δ_*mm*_, for *i* ≤ *j*, *i, j* = 1, …, *m* − 1.

Model (6) is an extension of the one-locus G2A model proposed in Zeng et al. (2005) to one QTL with multiple alleles. Note that no *v_ij_*(*g*)'s for *i* < *j* are needed in the bi-allelic case. The combined index variables *v_ii_*(*g*), *i* = 1, …, *m* − 1, are also slightly different from the ones defined in Zeng et al. (2005) by a scalar of (−2) folds. Here we drop the scalar (−2) so that the coefficient δ^*^_ii_ can keep the same interpretation as δ^*i^_*i_ in model (4). In addition, the combined index variables *v_ij_* (*i* < *j*) defined above are not exactly the same as the ones we suggested in the discussion section of Wang (2011). Based on the previously defined inheritance indicator variables, we define w^*^_i_ = *z_P_i__* + *z_M_i__* and v^*^_ij_(*g*) = *z_P_i__z_M_j__* + *z_P_j__z_M_i__* for *i* < *j*. Then here we have *v_ij_* = v^*^_ij_ − (p_i_w^*^_j_ + p_j_w^*^_i_) + 2*p_i_p_j_*, for *i* < *j*.

Still, model (6) retains the nice feature of the classical Fisher's model on partition of the genotypic variance. The additive variance component $V_{A}=V\left(\sum_{i\;=\;1}^{m\;-\;1}\alpha_{i}^{*}w_{i}(g)\right)$, which is contributed by the additive effects of both paternal and maternal alleles. The dominant variance component $V_{D}=V\left(\sum_{i\;=\;1}^{m\;-\;1}\delta_{ii}^{*}v_{ii}(g)+\sum_{i\;<\;j}\delta_{ij}^{*}v_{ij}(g)\right)$, which is contributed by all the interactions between paternal and maternal alleles. Under HWE, we have μ = *E*(*G*) and an orthogonal partition on the variance of the expected genotypic values *V*(*E*(*G*|*g*)) = *V_A_* + *V_D_*, where

$$\begin{array}{l} V_{A}=2\sum_{i\;=\;1}^{m\;-\;1}p_{i}{\left(\alpha_{i}^{*}\right)}^{2}-2{\left(\sum_{i\;=\;1}^{m\;-\;1}p_{i}\alpha_{i}^{*}\right)}^{2},\\ V_{D}=\sum_{i,j\;=\;1}^{m\;-\;1}p_{i}p_{j}{\left(\delta_{ij}^{*}\right)}^{2}-2\sum_{j\;=\;1}^{m\;-\;1}p_{j}\text{​}{\left(\sum_{i\;=\;1}^{m\;-\;1}p_{i}\delta_{ij}^{*}\text{​}\right)}^{2}+{\left(\sum_{i,j\;=\;1}^{m\;-\;1}p_{i}p_{j}\delta_{ij}^{*}\right)}^{2}\text{​​}. \end{array}$$

Here we define δ^*^_ji_ = δ^*^_ij_, for *i* < *j*. In HWD, the desired orthogonal partition on the variance of the expected genotypic values is no longer held. But the model can still allow us to capture the disequilibria via covariances between those index variables *x_P_i__*'s and *x_M_j__*'s (see **Appendix B**).

As an example, let us consider a QTL with 3 alleles *A*_1_, *A*_2_, and *A*_3_. By taking *A*_3_ as the baseline allele, model (6) leads to

(7) $$\begin{array}{l} E\left(G|g\right)=\mu +\alpha_{1}^{*}w_{1}(g)+\alpha_{2}^{*}w_{2}(g)+\delta_{11}^{*}v_{11}(g)+\delta_{22}^{*}v_{22}(g)\\ \;+\delta_{12}^{*}v_{12}(g). \end{array}$$

Or, in a matrix form, we have

$$\begin{array}{l} \text{​}\left(\text{​}\begin{array}{l} G_{11}\\ G_{12}\\ G_{22}\\ G_{13}\\ G_{23}\\ G_{33} \end{array}\text{​}\right)\text{​ }=\left(\begin{array}{llllll} 1 & 2(1-p_{1}) & -2p_{2} & {\left(1-p_{1}\right)}^{2} & p_{2}^{2} & -2p_{2}(1-p_{1})\\ 1 & 1-2p_{1} & 1-2p_{2} & -p_{1}(1-p_{1}) & -p_{2}(1-p_{2}) & 1-p_{1}-p_{2}+2p_{1}p_{2}\\ 1 & -2p_{1} & 2(1-p_{2}) & p_{1}^{2} & {\left(1-p_{2}\right)}^{2} & -2p_{1}(1-p_{2})\\ 1 & 1-2p_{1} & -2p_{2} & -p_{1}(1-p_{1}) & p_{2}^{2} & -p_{2}(1-2p_{1})\\ 1 & -2p_{1} & 1-2p_{2} & p_{1}^{2} & -p_{2}(1-p_{2}) & -p_{1}(1-2p_{2})\\ 1 & -2p_{1} & -2p_{2} & p_{1}^{2} & p_{2}^{2} & 2p_{1}p_{2} \end{array}\right)\\ \;\left(\begin{array}{r} \mu \\ \alpha_{1}^{*}\\ \alpha_{2}^{*}\\ \delta_{11}^{*}\\ \delta_{22}^{*}\\ \delta_{12}^{*} \end{array}\right) \end{array}$$

If we choose *A*_1_ (or *A*_2_) instead of *A*_3_ as the baseline allele, we can obtain different re-parameterizations of the six expected genotypic values. But they all give the same partition on the variance of the expected genotypic values. Álvarez-Castro and Yang (2011) also presented a similar re-parameterization of the six expected genotypic values from their NOIA model. It appears that their one-locus 3-allele NOIA model and the GMA model (7) above are likely equivalent on partitioning the variance of the six expected genotypic values, as we will see later in the Example Subsection 2.4.

Similar to the phase-known case, we can estimate the additive, dominance variance components and the covariance Cov(*A, D*) from a random sample when the QTL genotypes are available. First, we can compute the values of the combined index variables *w_i_(g_k_)* and *v_ij_(g_k_)* at the target QTL for each subject *k* = 1, …, *n*. Next, we can incorporate model (6) into a regression model with other possible adjusted covariates. By treating those combined index variables as regular fixed covariates, we can fit the regression model using the standard LS approach. Then, based on the fitted model, we compute the additive and dominance components $A(k)=\sum_{i\;=\;1}^{m\;-\;1}{\hat{\alpha }}_{i}^{*}w_{i}(g_{k})$ and $D(k)=\sum_{i\;=\;1}^{m\;-\;1}{\hat{\delta }}_{ii}^{*}v_{ii}(g_{k})+\sum_{i\;<\;j}{\hat{\delta }}_{ij}^{*}v_{ij}(g_{k})$ for each subject *k* = 1, …, *n*, where ${\hat{\alpha }}_{i}^{*},\;{\hat{\delta }}_{ii}^{*}$ and ${\hat{\delta }}_{ij}^{*}$ are LSE of the model parameters. Finally, we can estimate *V_A_* and *V_D_* as the sample variances of {*A*(*k*), *k* = 1, …, *n*} and {*D*(*k*), *k* = 1, …, *n*}, respectively. Meanwhile, an estimate of Cov(*A, D*) is given by the sample covariance between {*A*(*k*), *k* = 1, …, *n*} and {*D*(*k*), *k* = 1, …, *n*}.

### 2.3. Multi-locus models

The one-locus GMA models can be extended to multiple loci. Typically, for each locus *k*, we can create the mean-corrected index variables *x*^(*k*)^*_P_i__*, *x*^(*k*)^*_M_j__* (or combined index variables *w_k,i_, v_k,ij_* for phase-unknown genotypes) in the same way as in the one-locus case. Then we can build a multi-locus GMA model by including the within locus additive and dominance effects as well as the locus-by-locus interactions (i.e., epistases). The proposed multi-locus GMA model allows LD among multiple loci even though the LD may affect the partition on the variance of the expected genotypic values.

Consider *L* loci with alleles *A*_*k*1_, …, *A_km_k__* at the *k*-th locus for *k* = 1, …, *L*. With phase known, there are totally *m*^2^_1_··· *m*^2^_*L*_ possible expected genotypic values: *G*^*s*_1_···*s_L_*^_*t*_1_···*t*_*L*__ = *E*[*G*|*g* = (*A*_1*s*_1__···*A_Ls_L__*, *A*_1*t*_1__···*A_Lt_L__*)], where the joint genotype (*A*_1*s*_1__···*A_Ls_L__*, *A*_1*t*_1__···*A_Lt_L__*) is formed by the union of a paternal gamete *A*_1*s*_1__···*A_Ls_L__* and a maternal gamete *A*_1*t*_1__···*A_Lt_L__* with *s_k_, t_k_* ∈ {1, …, *m_k_*} for *k* = 1, …, *L*. Let *p^P^_ks_k__* and *p^M^_kt_k__* be the frequencies of the paternal allele *A_ks_k__* and maternal allele *A_kt_k__*, respectively. At each locus *k* = 1, …, *L*, we define the mean-corrected index variables *x*^(*k*)^*_Ps_k__* for paternal alleles *A*_ks_k__ (*s_k_* = 1, …, *m_k_*) and *x*^(*k*)^*_Mt_k__* for maternal alleles *A_kt_k__* (*t_k_* = 1, …, *m_k_*) in the same way as in the one-locus case. Then, when we choose *A*_1*m*_1__, …, *A_Lm_L__* as the baseline alleles, a fully parameterized *L*-locus GMA model can be expressed as

(8) $$\begin{array}{l} \text{​​​}E(G|g)=\sum_{i_{1}\;=\;0}^{1}\sum_{j_{1}\;=\;0}^{1}\cdots \sum_{i_{L}\;=\;0}^{1}\overset{1}{\underset{j_{L}\;=\;0}{\sum }}\sum_{\left\{s_{1},\text{if}\;i_{1}\;=\;1\right\}}\sum_{\left\{t_{1},\text{if}\;j_{1}\;=\;1\right\}}\text{​}\text{​}\cdots \sum_{\left\{s_{L},\text{if}\;i_{L}\;=\;1\right\}}\text{​}\text{​​​​​​​​}\\ \;\sum_{\left\{t_{L},\text{if}\;j_{L}\;=\;1\right\}}\text{​}\text{​}\alpha_{t_{1}^{*}\cdots t_{L}^{*}}^{s_{1}^{*}\cdots s_{L}^{*}}\cdot {\left(x_{Ps_{1}}^{\left(1\right)}\right)}^{i_{1}}{\left(x_{Mt_{1}}^{\left(1\right)}\right)}^{j_{1}}\cdots {\left(x_{Ps_{L}}^{\left(L\right)}\right)}^{i_{L}}{\left(x_{Mt_{L}}^{\left(L\right)}\right)}^{j_{L}}\text{​​​}\;\;\;\;\; \end{array}$$

where the summation of *s_k_* (or *t_k_*) is from 1 up to (*m_k_* − 1) for *k* = 1, …, *L*; *s_k_* (or *t_k_*) refers to a particular paternal allele *A_ks_k__* (or maternal allele *A_kt_k__*) at the *k*-th locus; and *i_k_* (or *j_k_*) is an indicator variable for the presence or absence of a paternal (or maternal) allele at locus *k* which is involved in a term. The coefficient α^s^*^_1_···s^*^_L_^_t^*^_1_···t^*^_L__ in each term represents an average allelic effect of a single paternal or maternal allele, or an allelic interaction from a set of paternal and maternal alleles that are involved in this term with respect to the baseline alleles *A*_1*m*_1__, …, *A_Lm_L__*. The superscripts (or subscripts) in α^s^*^_1_···s^*^_L_^_t^*^_1_···t^*^_L__ are defined as s^*^_k_ = *i_k_* · *s_k_* (or t^*^_k_ = *j_k_* · *t_k_*), indicating which paternal (or maternal) allele at locus *k* is involved in this term. If the term does not have any paternal (or maternal) allele at locus *k* being involved, then we have s^*^_k_ = 0 (or t^*^_k_ = 0). Note that $na_{P}=\sum_{k\;=\;1}^{L}i_{k}$ (or $na_{M}=\sum_{k\;=\;1}^{L}j_{k}$) specifies the total number of paternal (or maternal) alleles being involved in a term. We refer the total number of both paternal and maternal alleles that are involved in a term *na* = *na_P_* + *na_M_* as the order of this term.

The multi-locus GMA model (8) provides a full re-parameterization of the *m*^2^_1_···*m*^2^_*L*_ expected genotypic values *G*^*s*_1_···*s_L_*^_*t*_1_···*t_L_*_ with phase-known genotypes without using redundant parameters. Note that *E*(*G*|*g*) can also be partitioned into a sum of the following genetic components

$$\begin{array}{l} C_{j_{1}\cdots j_{L}}^{i_{1}\cdots i_{L}}=\sum_{\left\{s_{1},\text{if}\;i_{1}\;=\;1\right\}}\sum_{\left\{t_{1},\text{if}\;j_{1}\;=\;1\right\}}...\sum_{\left\{s_{L},\text{if}\;i_{L}=1\right\}}\sum_{\left\{t_{L},\text{if}\;j_{L}=1\right\}}\\ \;\alpha_{t_{1}^{*}\cdots t_{L}^{*}}^{s_{1}^{*}\cdots s_{L}^{*}}\cdot {\left(x_{Ps_{1}}^{\left(1\right)}\right)}^{i_{1}}{\left(x_{Mt_{1}}^{\left(1\right)}\right)}^{j_{1}}\cdots {\left(x_{Ps_{L}}^{\left(L\right)}\right)}^{i_{L}}{\left(x_{Mt_{L}}^{\left(L\right)}\right)}^{j_{L}}, \end{array}$$

where *i*_1_, *j*_1_, …, *i_L_, j_L_* = 0, 1 are indicator variables, which specify the parental origins of the contributing alleles. In other words, each genetic component consists of the terms with the same order and having their alleles all coming from the same subset of loci with the same parental origins but allowing varied allelic types. The classical genetic variance components can then be defined as variances of these genetic components. Note that the component *C*^0···0^_0···0_ is a constant, which corresponds to an intercept without any alleles being involved. Therefore, for a *L*-locus GMA model with phase known genotypes, there are in total (2^2*L*^−1) genetic variance components $V\left(C_{j_{1}\cdots j_{L}}^{i_{1}\cdots i_{L}}\right)$, where *i*_1_, *j*_1_, …, *i_L_, j_L_* = 0, 1 but not all being zeros. Among them, there are *L* paternal and *L* maternal variance components of order 1 with each having a single paternal or maternal allele being involved, *L* dominance variance components of order 2 with each having two alleles within the same locus being involved, *L*(*L* − 1)/2 paternal by paternal (or maternal by maternal) variance components of order 2 with two paternal (or maternal) alleles coming from different loci, and *L*(*L* − 1) paternal by maternal variance components of order 2 with one paternal and one maternal allele coming from two different loci. For examples, the within-locus paternal, maternal and dominance variance components at a locus *k* can be written as

$$\begin{array}{l} V_{A_{P}^{\left(k\right)}}=V\left(\sum_{s_{k}\;=\;1}^{m_{k}\;-\;1}\alpha_{0\cdots 0\cdots 0}^{0\cdots s_{k}\cdots 0}\cdot x_{Ps_{k}}^{\left(k\right)}\right)=V\left(C_{0\cdots 0\cdots 0}^{0\cdots 1\cdots 0}\right),\\ V_{A_{M}^{\left(k\right)}}=V\left(\sum_{t_{k}\;=\;1}^{m_{k}\;-\;1}\alpha_{0\cdots t_{k}\cdots 0}^{0\cdots 0\cdots 0}\cdot x_{Mt_{k}}^{\left(k\right)}\right)=V\left(C_{0\cdots 1\cdots 0}^{0\cdots 0\cdots 0}\right),\\ V_{D^{\left(k\right)}}=V\left(\sum_{s_{k}\;=\;1}^{m_{k}\;-\;1}\sum_{t_{k}\;=\;1}^{m_{k}\;-\;1}\alpha_{0\cdots t_{k}\cdots 0}^{0\cdots s_{k}\cdots 0}\cdot x_{Ps_{k}}^{\left(k\right)}x_{Mt_{k}}^{\left(k\right)}\right)=V\left(C_{0\cdots 1\cdots 0}^{0\cdots 1\cdots 0}\right), \end{array}$$

respectively (*k* = 1, …, *L*). For a pair of loci *j* ≠ *k*, the two-locus paternal by paternal variance component is

$$\begin{array}{l} V_{A_{P}^{\left(j\right)}\times A_{P}^{\left(k\right)}}=V\left(\sum_{s_{j}\;=\;1}^{m_{j}\;-\;1}\sum_{s_{k}\;=\;1}^{m_{k}\;-\;1}\alpha_{0\cdots 0\cdots 0\cdots 0}^{0\cdots s_{j}\cdots s_{k}\cdots 0}\cdot x_{Ps_{j}}^{\left(j\right)}x_{Ps_{k}}^{\left(k\right)}\right)\\ \;=V\left(C_{0\cdots 0\cdots 0\cdots 0}^{0\cdots 1\cdots 1\cdots 0}\right), \end{array}$$

and the two-locus paternal by maternal variance component is

$$\begin{array}{l} V_{A_{P}^{\left(j\right)}\times A_{M}^{\left(k\right)}}=V\left(\sum_{s_{j}\;=\;1}^{m_{j}\;-\;1}\sum_{t_{k}\;=\;1}^{m_{k}\;-\;1}\alpha_{0\cdots 0\cdots t_{k}\cdots 0}^{0\cdots s_{j}\cdots 0\cdots 0}\cdot x_{Ps_{j}}^{\left(j\right)}x_{Mt_{k}}^{\left(k\right)}\right)\\ \;=V\left(C_{0\cdots 0\cdots 1\cdots 0}^{0\cdots 1\cdots 0\cdots 0}\right). \end{array}$$

The variance component of epistases with the highest order is

$$\begin{array}{l} \text{​​​​}V_{D^{\left(1\right)}\times \cdots \times D^{\left(L\right)}}\\ \;=V\left(\sum_{s_{1}\;=\;1}^{m_{1}\;-\;1}\sum_{t_{1}\;=\;1}^{m_{1}\;-\;1}\cdots \sum_{s_{L}\;=\;1}^{m_{L}\;-\;1}\sum_{t_{L}\;=\;1}^{m_{L}\;-\;1}\alpha_{t_{1}\cdots t_{L}}^{s_{1}\cdots s_{L}}x_{Ps_{1}}^{\left(1\right)}x_{Mt_{1}}^{\left(1\right)}\cdots x_{Ps_{L}}^{\left(L\right)}x_{Mt_{L}}^{\left(L\right)}\right)\\ \;=V(C_{1\cdots 1}^{1\cdots 1}), \end{array}$$

which has 2*L* alleles being involved with two alleles per locus.

Based on these genetic components, we can partition the variance of the expected genotypic values into the variances and covariances of the genetic components. Still, the coefficients α^s^*^_1_···s^*^_L_^_t^*^_1_···t^*^_L__ are defined based on the baseline alleles *A*_1*m*_1__, …, *A_Lm_L__*. But the variances and covariances of these genetic components do not depend on the choice of the baseline alleles. Under HWE, the within-locus paternal index variables {*x*^(*k*)^*_Ps_k__, s_k_* = 1, …, *m_k_*} are independent of the maternal index variables {*x*^(*k*)^*_Mt_k__, t_k_* = 1, …, *m_k_*} for each *k* = 1, …, *L*. With LE, the index variables {*x*^(*j*)^*_Ps_j__, x*^(*j*)^*_Mt_j__, s_j_, t_j_* = 1, …, *m_j_*} at a locus *j* are also independent of the index variables {*x*^(*k*)^*_Ps_k__, x*^(*k*)^*_Mt_k__, s_k_, t_k_* = 1, …, *m_k_*} at a different locus *k* (*k* ≠ *j*). To achieve an orthogonal partition on the variance of the expected genotypic values, we need to further assume that all the paternal index variables {*x*^(*k*)^*_Ps_k__, s_k_* = 1, …, *m_k_, k* = 1, …, *L*} are independent of the maternal index variables {*x*^(*k*)^*_Mt_k__, t_k_* = 1, …, *m_k_, k* = 1, …, *L*} across all the loci; i.e., the so-called gametic equilibrium (see Wang and Zeng, 2006). Under both the gametic and linkage equilibria, each genetic component has its mean $E\left(C_{j_{1}\cdots j_{L}}^{i_{1}\cdots i_{L}}\right)=0$, and the covariances between different genetic components are zeros. Thus, we have *E*(*G*) = α^0···0^_0···0_ and an orthogonal partition on the variance of the expected genotypic values is given by

(9) $$V(E(G|g))=\sum_{i_{1}\;=\;0}^{1}\sum_{j_{1}\;=\;0}^{1}\cdots \sum_{i_{L}\;=\;0}^{1}\sum_{j_{L}\;=\;0}^{1}V\left(C_{j_{1}\cdots j_{L}}^{i_{1}\cdots i_{L}}\right),$$

where

$$\begin{array}{l} \text{​​​​}V\left(C_{j_{1}\cdots j_{L}}^{i_{1}\cdots i_{L}}\right)=\sum_{\left\{s_{1},{s^{\prime }}_{1},\text{if}\;i_{1}\;=\;1\right\}}{\left(p_{1s_{1}}^{P}1_{\left\{s_{1}\;=\;{s^{\prime }}_{1}\right\}}-p_{1s_{1}}^{P}p_{1{s^{\prime }}_{1}}^{P}\right)}^{i_{1}}\\ \;\sum_{\left\{t_{1},{t^{\prime }}_{1},\text{if}\;j_{1}\;=\;1\right\}}{\left(p_{1t_{1}}^{M}1_{\left\{t_{1}\;=\;{t^{\prime }}_{1}\right\}}-p_{1t_{1}}^{M}p_{1{t^{\prime }}_{1}}^{M}\right)}^{\;j_{1}}\cdots \\ \;\sum_{\left\{s_{L},{s^{\prime }}_{L},\text{if}\;i_{L}\;=\;1\right\}}{\left(p_{Ls_{L}}^{P}1_{\left\{s_{L}\;=\;{s^{\prime }}_{L}\right\}}-p_{Ls_{L}}^{P}p_{L{s^{\prime }}_{L}}^{P}\right)}^{i_{L}}\\ \;\sum_{\left\{t_{L},{t^{\prime }}_{L},\text{if}\;j_{L}\;=\;1\right\}}{\left(p_{Lt_{L}}^{M}1_{\left\{t_{L}\;=\;{t^{\prime }}_{L}\right\}}-p_{Lt_{L}}^{M}p_{L{t^{\prime }}_{L}}^{M}\right)}^{j_{L}}\cdot \alpha_{t_{1}^{*}\cdots t_{L}^{*}}^{s_{1}^{*}\cdots s_{L}^{*}}\alpha_{{t^{\prime }}_{1}^{*}\cdots {t^{\prime }}_{L}^{*}}^{{s^{\prime }}_{1}^{*}\cdots {s^{\prime }}_{L}^{*}}. \end{array}$$

Similarly, we can construct multi-locus GMA models for QTL with phase unknown genotypes. Without distinguishing the parental origin of the alleles, there are totally ∏^*L*^_*k* = 1 _*m_k_*(*m_k_* + 1)/2^*L*^ possible expected genotypic values: *G*_*s*_1_*t*_1_···*s*_*L*_*t*_*L*__ = *E*[*G*(*g*)|*g* = (*A*_1*s*_1__*A*_1*t*_1__, …, *A_Ls_L__A_Lt_L__*)], for *s_k_, t_k_* = 1, …, *m_k_* and *k* = 1, …, *L*. We assume that the paternal and maternal allele frequencies are the same and denoted by *p_ks_k__*. We define the combined index variables *w_k, i_*(*g*) and *v_k,ij_* at each locus *k* in the same way as the one-locus case for *k* = 1, …, *L*. Then, when we choose *A*_1*m*_1__, …, *A_Lm_L__* as the baseline alleles, a fully parameterized *L*-locus GMA model for QTL with phase unknown genotypes can be expressed as

(10) $$\begin{array}{l} E(G|g)=\sum_{i_{1}\;=\;0}^{2}\cdots \sum_{i_{L}\;=\;0}^{2}\sum_{\left\{s_{1},\text{if}\;i_{1}\;=\;1\right\}}\cdots \\ \sum_{\left\{s_{L},\text{if }i_{L}=1\right\}}\sum_{\left\{s_{1}\leq t_{1},\text{if}\;i_{1}=2\right\}}...\sum_{\left\{s_{L}\leq t_{L},\text{if}\;i_{L}=2\right\}}\\ \;\alpha_{s_{1}^{*}\cdots s_{L}^{*}}\cdot \left(w_{1,s_{1}}^{1_{\left\{i_{1}=1\right\}}}\cdot v_{1,s_{1}t_{1}}^{1_{\left\{i_{1}=2\right\}}}\right)\cdots \left(w_{L,s_{L}}^{1_{\left\{i_{L}=1\right\}}}\cdot v_{L,s_{L}t_{L}}^{1_{\left\{i_{L}=2\right\}}}\right), \end{array}$$

where 1_{*i_k_=j*}_ is the Kronecker function which equals 1 when *i_k_* = *j* and 0 otherwise, for *k* = 1, …, *L* and *j* = 1 or 2; the summation of *s_k_* (or *t_k_*) is from 1 up to *m_k_* − 1 with *s_k_* (or *t_k_*) referring to allele *A_ks_k__* (or *A_kt_k__*) at a locus *k* (*k* = 1, …, *L*); and *i_k_* specifies how many alleles at a locus *k* are involved in this term. If *i_k_* = 1, this term has only one allele *A_ks_k__* (either paternal or maternal) being involved via *w_k,s_k__* and we set s_k_^*^ = *s_k_*. If *i_k_* = 2, both the paternal and maternal alleles *A_ks_k__* and *A_kt_k__* are involved via *v_k,s_k_t_k__* in this term and we set s^*^_k_ = *s_k_t_k_* regardless of the order of *s_k_* and *t_k_*. When *i_k_* = 0, this term does not have any alleles at locus *k* being involved and we have $\left(w_{k,s_{k}}^{1_{\left\{i_{k}=1\right\}}}\cdot v_{k,s_{k}t_{k}}^{1_{\left\{i_{k}=2\right\}}}\right)=1$. The coefficient α_s^*^_1_···s^*^_L__ represents the average allelic effect of a single allele, or an allelic interaction from all the alleles that are involved in this term, with respect to the baseline alleles *A*_1*m*_1__, …, *A_Lm_L__*. We still refer the total number of alleles being involved in each term; i.e., $na=\sum_{k\;=\;1}^{L}i_{k}$, as the order of this term.

Based on the above model, we can define the genetic components as

$$\begin{array}{l} C_{i_{1}\cdots i_{L}}=\sum_{\left\{s_{1},\text{if}\;i_{1}\;=\;1\right\}}...\sum_{\left\{s_{L},\text{if}\;i_{L}\;=\;1\right\}}\sum_{\left\{s_{1}\;\leq \;t_{1},\text{if}\;i_{1}\;=\;2\right\}}...\sum_{\left\{s_{L}\;\leq \;t_{L},\text{if}\;i_{L}\;=\;2\right\}}\\ \;\alpha_{s_{1}^{*}\cdots s_{L}^{*}}\cdot \left(w_{1,s_{1}}^{1_{\left\{i_{1}=1\right\}}}\cdot v_{1,s_{1}t_{1}}^{1_{\left\{i_{1}=2\right\}}}\right)\cdots \left(w_{L,s_{L}}^{1_{\left\{i_{L}=1\right\}}}\cdot v_{L,s_{L}t_{L}}^{1_{\left\{i_{L}=2\right\}}}\right), \end{array}$$

for *i*_1_, …, *i_L_* = 0, 1, 2, with each component being a summation of the terms with their alleles all coming from the same subset of loci and having the same number of alleles from each locus. Excluding the one with *i*_1_ = ··· = *i_L_* = 0, which corresponds to an intercept, there are in total 3^*L*^ − 1 genetic variance components *V*(*C*_*i*_1_···*i_L_*_) for *i*_1_, …, *i_L_* = 0, 1, 2. Among them, there are *L* additive variance components with each having a single paternal or maternal allele being involved, *L* dominance variance components with each having both paternal and maternal alleles within the same locus being involved, *L*(*L* − 1)/2 additive by additive variance components with two alleles coming from different loci, etc. For examples, the within-locus additive and dominance variance components at a locus *k* can be written as

$$\begin{array}{l} V_{A^{\left(k\right)}}=V\left(\sum_{s_{k}\;=\;1}^{m_{k}\;-\;1}\alpha_{0\cdots s_{k}\cdots 0}\cdot w_{k,s_{k}}\right)=V(C_{0\cdots 1\cdots 0}),\\ V_{D^{\left(k\right)}}=V\left(\sum_{s_{k}\;\leq \;t_{k}}\alpha_{0\cdots s_{k}t_{k}\cdots 0}\cdot v_{k,s_{k}t_{k}}\right)=V(C_{0\cdots 2\cdots 0}), \end{array}$$

respectively, for *k* = 1, …, *L*. For a pair of loci *j* ≠ *k*, the two-locus additive by additive interaction is

$$\begin{array}{l} V_{A^{\left(j\right)}\times A^{\left(k\right)}}=V\left(\sum_{s_{j}\;=\;1}^{m_{j}\;-\;1}\sum_{s_{k}\;=\;1}^{m_{k}\;-\;1}\alpha_{0\cdots s_{j}\cdots s_{k}\cdots 0}\cdot w_{j,s_{j}}w_{k,s_{k}}\right)\\ \;=V(C_{0\cdots 1\cdots 1\cdots 0}). \end{array}$$

The variance component of epistases with the highest order of 2*L* is given by

$$\begin{array}{l} \text{​​​​}V_{D^{\left(1\right)}\times \cdots \times D^{\left(L\right)}}\\ \;=V\left(\sum_{s_{1}\;\leq \;t_{1}}\cdots \sum_{s_{L}\;\leq \;t_{L}}\alpha_{s_{1}t_{1},\cdots ,s_{L}t_{L}}v_{1,s_{1}t_{1}}\cdots v_{L,s_{L}t_{L}}\right)\\ \;=V(C_{2\cdots 2}). \end{array}$$

Under both the gametic and linkage equilibria, we have *E*(*G*) = α_0···0_ and an orthogonal partition on the variance of the expected genotypic values

(11) $$V(E(G|g))=\sum_{i_{1}\;=\;0}^{2}\cdots \sum_{i_{L}\;=\;0}^{2}V(C_{i_{1}\cdots i_{L}}),$$

where

$$\begin{array}{l} \text{​​​​}V(C_{i_{1}\cdots i_{L}})=\sum_{\left\{s_{1},{s^{\prime }}_{1},\text{if}\;i_{1}=1\right\}}{\left\{2\left(p_{1s_{1}}1_{\left\{s_{1}={s^{\prime }}_{1}\right\}}-p_{1s_{1}}p_{1s_{1}}\right)\right\}}^{1_{\left\{i_{1}=1\right\}}}\cdots \\ \sum_{\left\{s_{L},{s^{\prime }}_{L},\text{if}\;i_{L}=1\right\}}{\left\{2\left(p_{Ls_{L}}1_{\left\{s_{L}={s^{\prime }}_{L}\right\}}-p_{Ls_{L}}p_{L{s^{\prime }}_{L}}\right)\right\}}^{1_{\left\{i_{L}=1\right\}}}\\ \sum_{\left\{s_{1},{s^{\prime }}_{1},t_{1},{t^{\prime }}_{1},\text{if}\;i_{1}=2\right\}}\{\left(p_{1s_{1}}1_{\left\{s_{1}={s^{\prime }}_{1}\right\}}-p_{1s_{1}}p_{1{s^{\prime }}_{1}}\right)\\ \;{\left(p_{1t_{1}}1_{\left\{t_{1}={t^{\prime }}_{1}\right\}}-p_{1t_{1}}p_{1{t^{\prime }}_{1}}\right)\}}^{1_{\left\{i_{1}=2\right\}}}\cdots \\ \sum_{\left\{s_{L},{s^{\prime }}_{L},t_{L},{t^{\prime }}_{L},\text{if}\;i_{L}=2\right\}}\{\left(p_{Ls_{L}}1_{\left\{s_{L}={s^{\prime }}_{L}\right\}}-p_{Ls_{L}}p_{L{s^{\prime }}_{L}}\right)\\ \;{\left(p_{Lt_{L}}1_{\left\{t_{L}={t^{\prime }}_{L}\right\}}-p_{Lt_{L}}p_{L{t^{\prime }}_{L}}\right)\}}^{1_{\left\{i_{L}=2\right\}}}\alpha_{s_{1}^{*}\cdots s_{L}^{*}}\alpha_{s_{1}^{*}{}^{\prime }\cdots s_{L}^{*}{}^{\prime }}. \end{array}$$

Here, when *i_k_* = 2, we have s^*^_k_ = *s_k_t_k_* and s^*^′_*k*_ = *s′_k_t′_k_*. We also define α_s^*^_1_···s^*^_L__ (or α_s^*^′_1_···s^*^′_L__) to be the same if we switch the order of *s_k_* and *t_k_* in s^*^_k_ (or *s_k_*' and *t_k_*' in s^*^′_k_) for *k* = 1, …, *L*. In the presence of disequilibria, the desired orthogonal partition may no longer hold. However, regardless of the equilibrium, the coefficients α_s^*^_1_···s^*^_L__ in model (10) are defined based on the baseline alleles *A*_1*m*_1__, …, *A_Lm_L__*, while the variances and covariances of the genetic components do not depend on the choice of these baseline alleles.

In practice, we do not have to rely on the derived formula to estimate the genetic variance or covariance components. Similar to the one-locus case, given the observed QTL genotypes for a random sample from a study population, we can always incorporate model (8) or (10) into a regression model with other possible adjusted covariates and fit the model using standard LS approach. Then we can estimate various genetic variance components as well as the covariances among different genetic components based on the fitted model. A good fit of a fully parameterized GMA model often requires that the expected genotypic values for all possible joint genotypes of the QTL are estimable from the study sample. If certain genotypes are not observable or rarely present in subjects from the study sample, a situation which likely happens when the number of alleles or the number of QTL is large with moderate or small sample size, the design matrix for the genetic effects could become singular which implies that some genetic variance components cannot be estimated reliably. But we do not have to use fully parameterized GMA models to model the expected genotypic values. In this case, we may want to build a reduced GMA model that can provide a good approximation to the expected genotypic values overall and meanwhile has a less complicated model structure. The fact that two terms within the same genetic component are unavoidably correlated suggests that we should perhaps treat each genetic component as a whole and keep or drop its terms all at once in building a GMA model. As genetic components of lower orders tend to have bigger impact on the expected genotypic values than the higher order ones, one way to construct a reduced GMA model is perhaps to go through a stepwise forward selection procedure by hierarchically adding the lowest order genetic component that can achieve a nominal significance level (e.g., 5%) but has not yet been selected in the model into the model one at a time. Here, the classical likelihood ratio statistic can be used to assess each genetic component for entering into or dropping from the model.

It has been known that the model building procedure is often sensitive to potential confounding among the selected variables. A GMA model uses the mean-corrected index variables *x*^(*k*)^*_Ps_k__, x*^(*k*)^*_Mt_k__* (or their combined index variables *w_k,s_k__, v_k,s_k_t_k__* in the phase unknown case) as the basic units in constructing all its model terms. At least in an equilibrium population, these mean-corrected index variables can reduce the confounding among different genetic components and make them uncorrelated. This orthogonality implies that each genetic component can be assessed independently regardless of which components are presented in the model. On the other hand, an *F*_∞_ model can be thought of directly using the inheritance indicator variables *z*^(*k*)^*_Ps_k__* and *z*^(*k*)^*_Mt_k__* (or their merged genotype coding variables w^*^_k,s_k__(*g*) = *z*^(*k*)^*_Ps_k__* + *z*^(*k*)^*_Ms_k__* and v^*^_k,s_k_t_k__ = *z*^(*k*)^*_Ps_k__z*^(*k*)^*_Mt_k__* + *z*^(*k*)^*_Pt_k__z*^(*k*)^*_Ms_k__* in phase unknown case) as the basic units in constructing its terms (Wang and Zeng, 2009; Wang, 2011). Even in an equilibrium population, an *F*_∞_ model could have its low-order terms being highly confounded with other high-order terms when they contain shared alleles (see Zeng et al., 2005). As the result, in building a predictive model based on *F*_∞_ models, the stepwise forward selection procedure could be problematic because failing to include a significant higher order term (or component) in a reduced *F*_∞_ model could make the assessment of some low-order terms (or components) unreliable. On selecting significant QTL from a set of loci without having the locus-by-locus interactions being involved, the choice of using GMA or *F*_∞_ model in building a reduced model for the expected genotypic values should not matter much because using mean-corrected index variables mainly affects the intercept in this case. However, when we consider including epistases for a given set of QTL, the GMA model can appropriately use the orthogonal property among different genetic components to dissect the confounding at least in equilibrium populations, while it appears that the *F*_∞_ models cannot make full use of the equilibrium information. When disequilibria are present, as Hardy-Weinberg equilibria are expected to be held in most of the human genomic regions and LD mainly present for closely linked loci, we would expect that most of the genetic components in a GMA model are likely uncorrelated. Therefore, in most cases, using the GMA model could still be preferable to using *F*_∞_ model in building reduced models for expected genotypic values especially when epistases are involved.

### 2.4. Example

As an example, we apply the GMA model to a published experimental data set on the polymorphism at the human acid phosphatase locus (ACP1). The analysis of this data set was first conducted by Greene et al. (2000), and recently re-analyzed as an example in Álvarez-Castro and Yang (2011). The ACP1 gene involves 3 alleles A, B, C. Two phenotypic traits are considered, which measure the ACP1 enzyme activity (*y*^*ac*^) and inhibition (*y*^*in*^). The estimates of the expected genotypic values and the genotype frequencies are summarized in Table 1.

**Table 1:** **Expected genotypic values and observed genotype frequencies**.

	**ACP1 genotypes**					
	**AA**	**AB**	**BB**	**AC**	**BC**	**CC**
ACP1 enzyme activity (*G^ac^*)	122.4	153.9	188.3	183.6	212.3	240.0
ACP1 enzyme inhibition (*G^in^*)	41.2	37.9	34.4	58.7	53.1	76.0
Genotype frequency	0.1242	0.4139	0.3349	0.0445	0.0799	0.0025

From the genotype frequencies, we first estimate the allele frequencies as *p_A_* = 0.3534, *p_B_* = 0.5818, and *p_C_* = 0.0647. Then, for each trait, we fit a separate GMA model (7) to its expected genotypic values by taking *C* as the baseline allele and creating the combined index variables *w*_1_(*g*), *w*_2_(*g*), *v*_11_(*g*), *v*_22_(*g*), *v*_12_(*g*). For ACP1 enzyme activity, we obtain LSE of the model parameters as μ = 167.735, α^*^_1_ = −59.260, α^*^_2_ = −26.254, δ^*^_11_ = −4.800, δ^*^_22_ = 3.700 and δ^*^_12_ = −2.000. For ACP1 enzyme inhibition, we have μ = 39.386, α^*^_1_ = −16.149, α^*^_2_ = −19.714, δ^*^_11_ = −0.200, δ^*^_22_ = 4.200, and δ^*^_12_ = 2.100.

Next, for each trait separately, we calculate *A(g_k_)* = α^*^_1_*w*_1_(*g_k_*) + α^*^_2_*w*_2_(*g_k_*) and *D*(*g_k_*) = δ^*^_11_*v*_11_(*g_k_*) + δ^*^_22_*v*_22_(*g_k_*) + δ^*^_12_*v*_12_(*g_k_*), *k* = 1, …, 6, for the six ACP1 genotypes. Based on the genotype frequencies, we then calculate the genetic variance components. For ACP1 enzyme activity, we obtain *V_A_* = 658.868, *V_D_* = 0.973 and the covariance Cov(*A, D*) = −0.356. The total variance of the expected genotypic values is *V*(*E*(*G^ac^*|*g*)) = 659.129, and *V_A_*/*V*(*E*(*G^ac^*|*g*)) = 99.96%. For ACP1 enzyme inhibition, we have *V_A_* = 44.920, *V_D_* = 0.146 and the covariance Cov(*A, D*) = −0.217. The total variance of the expected genotypic values is *V*(*E*(*G^in^*|*g*)) = 44.632, and *V_A_*/*V*(*E*(*G^in^*|*g*)) = 100.65%. Note that the partition *V*(*E*(*G*|*g*)) = *V_A_* + *V_D_* + 2Cov(*A, D*), which is not orthogonal for both traits due to a slight deviation from HWE at ACP1 locus as we have observed from the genotype frequencies in Table 1. It is also interesting to see that for ACP1 enzyme inhibition, *V_A_*/*V*(*E*(*G^in^*|*g*)) is bigger than 100% due to HWD and the fact that *V_D_* + 2Cov(*A, D*) < 0.

Using the same allele frequencies but assuming HWE, we would have slightly different genotype frequencies. Note that the LSE of the model parameters keep the same and do not depend on the genotype frequencies because they are completely determined by the allele frequencies and the six expected genotypic values when a fully-parameterized GMA is used. But the total variance of the expected genotypic values and its variance components will be different. For ACP1 enzyme activity, we obtain *V_A_* = 660.588, *V_D_* = 0.971, Cov(*A, D*) = 0.006, *V*(*E*(*G^ac^*|*g*)) = 661.573 and *V_A_*/*V*(*E*(*G^ac^*|*g*)) = 99.85%. For ACP1 enzyme inhibition, we have *V_A_* = 46.422, *V_D_* = 0.152, Cov(*A, D*) = 0.001, *V*(*E*(*G^in^*|*g*)) = 46.573 and *V_A_*/*V*(*E*(*G*^*in*^|*g*)) = 99.68%. The minor deviations from orthogonal partitions are likely due to some numerical round-off and the fact that the summation of the three allele frequencies is 0.9999 instead of exactly 1.

For this three-allele example, we also applied the NOIA model using the formulas (10) and (11) provided in Álvarez-Castro and Yang (2011), and in both HWE and HWD cases we obtained exactly the same results as above based on our own calculation, which however appear to be slightly different from what had been reported in Álvarez-Castro and Yang (2011). For example, based on the observed genotype frequencies, an estimate of *V_D_* = 1.15 for the trait of ACP1 enzyme inhibition was reported in Álvarez-Castro and Yang (2011), which is noticeably larger than *V_D_* = 0.146 that we obtain above.

## 3. Discussion

In the analysis of genetic variance components, a separation of the variations contributed by the additive allelic effects and allelic interactions is complicated by the fact that the observed genotypes are often phase-unknown. In this study, by appropriately merging the paternal and maternal allelic effects and allelic interactions in the phase-known situation, we propose a way to construct one-locus and multi-locus GMA models on analysis of genetic variance components for QTL with multiple alleles. In the same way as building a G2A model, we construct a GMA model by first specifying its design matrix for the genetic effects via some mean-corrected index variables. As these mean-corrected index variables are well defined based on the observed genotypes and allele frequencies, they can be treated as regular covariates for coding QTL genotypes. These one-locus or multi-locus GMA models can then be incorporated into standard regression models with other possible adjusted covariates and fitted using standard LS approach. Based on the fitted models, we can further estimate the genetic variance and covariance components through the sample variances and covariances of various genetic components. As we have pointed out, these GMA models can be applied to equilibrium populations as well as populations in Hardy-Weinberg and/or linkage disequilibria. By using the full set or a low-order subset of the index variables (or genetic components), the GMA model allows us to make either full or reduced re-parameterization of the genotypic values. When some loci have phase known genotypes while other loci have phase unknown genotypes (a possible hypothetical situation), a mixed GMA model could also be constructed by adopting the same modeling strategy.

Sometimes we may want to perform hypothesis tests on the existence of certain genetic variance and covariance components. Note that the GMA models have allele frequencies being involved in their design matrices. As allele frequencies often need to be estimated from the genotype data, they could contribute another source of variation in the LSE ($\hat{\beta }$) of the model parameters as well as the genetic variance components. When the allele frequencies can be accurately estimated, we could simply treat them as fixed constants. When the residuals in a regression model do not depend on the allele frequency estimates, based on the linear model theory (see Ravishanker and Dey, 2002), $\hat{\beta }$ are known to be unbiased with its covariance matrix cov($\hat{\beta }$) = σ^2^E[(*X*′*X*)^−1^], where *X* is the design matrix of a GMA model. In this case, we can assess the existence of variance components by performing traditional hypothesis tests on $\hat{\beta }$. In general, we could also assess the existence of these genetic variance and covariance components through a bootstrap procedure. By repeatedly drawing random samples of the same size from the observed random sample with replacement, we can estimate the genetic variance and covariance components for each bootstrap sample and meanwhile assess the variances in estimates of these genetic variance and covariance components and test for their existence.

In genetic studies, QTL with missing genotypes is a common phenomenon. GMA model can be used to fit QTL with missing genotypes. Rather than excluding patients with missing QTL genotypes, we could treat “missing” as an allele although this strategy may induce potential bias as we assume that all the missing alleles have the same genetic effect. GMA models could also be applied incorporation with various imputation methods. In recent years, there has been a great deal of interest in developing methodologies for QTL mapping using recombinant intercrosses from multiple inbred lines. In this case, the putative QTL often have their locations and genotypes unknown. But the allele frequencies of QTL could probably be inferred from the study design and the QTL genotypes might be imputable from their neighboring genetic markers. How to apply GMA models to this type of experimental crosses for QTL mapping could be a research topic for further exploration.

In summary, the analysis of genetic variance components for multi-allele QTL has been challenging due to complex allelic interactions and locus-by-locus interactions. In this study, we thoroughly explored the architecture of one-locus and multi-locus GMA models with either phase known or unknown genotypes. Particularly, we described in detail the architecture of the multi-locus GMA model, and how the model terms can be grouped into various genetic components. Under equilibria populations, we also derived formulas for orthogonal partition of the genetic variance components, which could be useful for analytical assessment of the variance components. Comparing to the classical Fisher model, the GMA models can estimate the genetic variance and covariance components more conveniently via standard LS approach for either one or multiple QTL with multiple alleles, in equilibrium as well as disequilibrium populations.

### Conflict of interest statement

The author declares that the research was conducted in the absence of any commercial or financial relationships that could be construed as a potential conflict of interest.

## Appendix A. one-locus model for phase known – in Hardy-Weinberg disequilibrium

In HWD, we can represent the genotype frequencies as *P_(A_i_,A_j_)_* = *p^i^p_j_* + *D^i^_j_*, where *D^i^_j_* measures the departure from HWE with constraints $\sum_{i\;=\;1}^{m}D_{j}^{i}=0$ for *j* = 1, …, *m*, and $\sum_{j\;=\;1}^{m}D_{j}^{i}=0$ for *i* = 1, …, *m*. Note that *D*^*i*^_*j*_ = *P_(A_i_,A_j_)_* − *p*^*i*^*p*_*j*_ = Cov(*x_P_i__, x_M_j__*), we can show that $E(G)=\mu +\sum_{i\;=\;1}^{m\;-\;1}\sum_{j\;=\;1}^{m\;-\;1}\delta_{*j}^{*i}D_{j}^{i}$, and the variance of the expected genotypic values becomes

$$V(E(G|g))=V_{A_{P}}+V_{A_{M}}+V_{D}+2\text{Cov}(A_{P},A_{M})+2\text{Cov}(A_{P},D)+2\text{Cov}(A_{M},D)$$

where $A_{P}=\sum_{i\;=\;1}^{m\;-\;1}\alpha^{*i}x_{P_{i}}(g),\;A_{M}=\sum_{j\;=\;1}^{m\;-\;1}\alpha_{*j}x_{M_{j}}(g)$ and $D=\sum_{i\;=\;1}^{m\;-\;1}\sum_{j\;=\;1}^{m\;-\;1}\delta_{*j}^{*i}x_{P_{i}}(g)x_{M_{j}}(g)$. The formulas on calculating the additive variance components *V_A_P__* and *V_A_M__* are the same as in HWE case. But the formula for dominance variance component *V_D_* becomes

$$\begin{array}{l} V_{D}=\sum_{i,j\;=\;1}^{m\;-\;1}p^{i}p_{j}{\left(\delta_{*j}^{*i}\right)}^{2}-\sum_{i\;=\;1}^{m\;-\;1}p^{i}{\left(\sum_{j\;=\;1}^{m\;-\;1}p_{j}\delta_{*j}^{*i}\right)}^{2}-\sum_{j\;=\;1}^{m\;-\;1}p_{j}{\left(\sum_{i\;=\;1}^{m\;-\;1}p^{i}\delta_{*j}^{*i}\right)}^{2}+{\left(\sum_{i,j\;=\;1}^{m\;-\;1}p^{i}p_{j}\delta_{*j}^{*i}\right)}^{2}\\ \;+\sum_{i,j\;=\;1}^{m\;-\;1}{\left(\delta_{*j}^{*i}\right)}^{2}D_{ij}-2\sum_{i\;=\;1}^{m\;-\;1}\left(\sum_{j\;=\;1}^{m\;-\;1}p_{j}\delta_{*j}^{*i}\right)\left(\sum_{j\;=\;1}^{m\;-\;1}D_{j}^{i}\delta_{*j}^{*i}\right)-2\sum_{j\;=\;1}^{m\;-\;1}\left(\sum_{i\;=\;1}^{m\;-\;1}p^{i}\delta_{*j}^{*i}\right)\left(\sum_{i\;=\;1}^{m\;-\;1}D_{j}^{i}\delta_{*j}^{*i}\right)\\ \;+2\left(\sum_{i,j\;=\;1}^{m\;-\;1}p^{i}p_{j}\delta_{*j}^{*i}\right)\left(\sum_{i,j\;=\;1}^{m\;-\;1}D_{j}^{i}\delta_{*j}^{*i}\right)+2\sum_{i,s\;=\;1}^{m\;-\;1}p^{i}\left(\sum_{j\;=\;1}^{m\;-\;1}p_{j}\delta_{*j}^{*s}\right)\left(\sum_{j\;=\;1}^{m\;-\;1}D_{j}^{s}\delta_{*j}^{*i}\right)-{\left(\sum_{i,j\;=\;1}^{m\;-\;1}\delta_{*j}^{*i}D_{j}^{i}\right)}^{2} \end{array}$$

For the covariances, we have

$$\begin{array}{l} \text{Cov}(A_{P},A_{M})=\sum_{i,j\;=\;1}^{m\;-\;1}D_{j}^{i}\alpha^{*i}\alpha_{*j}\\ \text{ Cov}(A_{P},D)=\sum_{i,j\;=\;1}^{m\;-\;1}D_{j}^{i}\alpha^{*i}\delta_{*j}^{*i}-\left(\sum_{i\;=\;1}^{m\;-\;1}p^{i}\alpha^{*i}\right)\left(\sum_{j,s\;=\;1}^{m\;-\;1}D_{j}^{s}\delta_{*j}^{*s}\right)-\sum_{j\;=\;1}^{m\;-\;1}\left(\sum_{i\;=\;1}^{m\;-\;1}\alpha^{*i}D_{j}^{i}\right)\left(\sum_{s\;=\;1}^{m\;-\;1}p^{s}\delta_{*j}^{*s}\right)\\ \text{ Cov}(A_{M},D)=\sum_{i,j\;=\;1}^{m\;-\;1}D_{j}^{i}\alpha_{*j}\delta_{*j}^{*i}-\left(\sum_{j\;=\;1}^{m\;-\;1}p_{j}\alpha_{*j}\right)\left(\sum_{i,t\;=\;1}^{m\;-\;1}D_{t}^{i}\delta_{*t}^{*i}\right)-\sum_{i\;=\;1}^{m\;-\;1}\left(\sum_{j\;=\;1}^{m\;-\;1}\alpha_{*j}D_{j}^{i}\right)\left(\sum_{t\;=\;1}^{m\;-\;1}p_{t}\delta_{*t}^{*i}\right) \end{array}$$

As the paternal and maternal alleles are correlated in HWD, we will likely have non-zero covariances among *A_P_*, *A_M_* and *D*.

## Appendix B. one-locus model for phase unknown – in Hardy-Weinberg disequilibrium

In HWD, we can represent the genotype frequencies as *P_A_i_A_i__* = *p*^2^*_i_* + *D_ii_* for *j* = 1, …, *m*, and *P_A_i_A_j__* = 2*p_i_p_j_* + 2*D_ij_* for *i* ≠ *j*. Since *p_i_* = *P_A_i_A_i__* + ∑*_j≠i_P_A_i_A_j__*/2, we have $\sum_{i\;=\;1}^{m}D_{ij}=0$ for *j* = 1, …, *m*; $\sum_{j\;=\;1}^{m}D_{ij}=0$ for *i* = 1, …, *m*; and *D_ij_* = *D_ji_*. As the disequilibrium measures can be represented as *D_ij_* = Cov(*x_P_i__, x_M_j__*) for *i, j* = 1, …, *m*, we can show that $E(G)=\mu +\sum_{i\;=\;1}^{m\;-\;1}\delta_{ii}^{*}D_{ii}+2\sum_{j\;=\;2}^{m\;-\;1}\sum_{i\;<\;j}\delta_{ij}^{*}D_{ij}$, and the variance partition of the expected genotypic values *V*(*E*(*G*|*g*)) = *V_A_* + *V_D_* + 2Cov(*A, D*), where

$$\begin{array}{l} \;V_{A}=2\sum_{i\;=\;1}^{m\;-\;1}p_{i}{\left(\alpha_{i}^{*}\right)}^{2}-2{\left(\sum_{i\;=\;1}^{m\;-\;1}p_{i}\alpha_{i}^{*}\right)}^{2}+2\left(\sum_{i\;=\;1}^{m\;-\;1}D_{ii}{\left(\alpha_{i}^{*}\right)}^{2}+2\sum_{i\;<\;j}\alpha_{i}^{*}\alpha_{j}^{*}D_{ij}\right)\\ \;V_{D}=\sum_{i,j\;=\;1}^{m\;-\;1}p_{i}p_{j}{\left(\delta_{ij}^{*}\right)}^{2}-2\sum_{j\;=\;1}^{m\;-\;1}p_{j}{\left(\sum_{i\;=\;1}^{m\;-\;1}p_{i}\delta_{ij}^{*}\right)}^{2}+{\left(\sum_{i,j\;=\;1}^{m\;-\;1}p_{i}p_{j}\delta_{ij}^{*}\right)}^{2}+\sum_{i,j\;=\;1}^{m\;-\;1}D_{ij}{\left(\delta_{ij}^{*}\right)}^{2}-4\sum_{j\;=\;1}^{m\;-\;1}\left(\sum_{i\;=\;1}^{m\;-\;1}p_{i}\delta_{ij}^{*}\right)\left(\sum_{i\;=\;1}^{m\;-\;1}D_{ij}\delta_{ij}^{*}\right)\\ \;+2\left(\sum_{i,j\;=\;1}^{m\;-\;1}p_{i}p_{j}\delta_{ij}^{*}\right)\left(\sum_{i,j\;=\;1}^{m\;-\;1}D_{ij}\delta_{ij}^{*}\right)+2\sum_{i,s\;=\;1}^{m\;-\;1}p_{i}\left(\sum_{j\;=\;1}^{m\;-\;1}p_{j}\delta_{sj}^{*}\right)\left(\sum_{j\;=\;1}^{m\;-\;1}D_{sj}\delta_{ij}^{*}\right)-{\left(\sum_{i,j\;=\;1}^{m\;-\;1}D_{ij}\delta_{ij}^{*}\right)}^{2}\\ \text{Cov}(A,D)=2\sum_{i,j\;=\;1}^{m\;-\;1}D_{ij}\alpha_{i}^{*}\delta_{ij}^{*}-2\left(\sum_{i\;=\;1}^{m\;-\;1}p_{i}\alpha_{i}^{*}\right)\left(\sum_{s,j\;=\;1}^{m\;-\;1}D_{sj}\delta_{sj}^{*}\right)-2\sum_{j\;=\;1}^{m\;-\;1}\left(\sum_{i\;=\;1}^{m\;-\;1}\alpha_{i}^{*}D_{ij}\right)\left(\sum_{s\;=\;1}^{m\;-\;1}p_{s}\delta_{sj}^{*}\right) \end{array}$$

In HWD, the genotype frequencies can also be parameterized as *P_A_i_A_i__* = *p*^2^_*i*_ + *p_i_*(1 − *p_i_*)*f*, and *P_A_i_A_j__* = 2*p_i_p_j_*(1 − *f*) for *i* ≠ *j* (see Weir, 1996). This is a special case of the above parameterization with *D_ii_* = *p_i_*(1 − *p_i_*)*f* and *D_ij_* = −*p_i_p_j_f*, for *i* ≠ *j*.
